# Genetic Mapping of the Leaf Number above the Primary Ear and Its Relationship with Plant Height and Flowering Time in Maize

**DOI:** 10.3389/fpls.2017.01437

**Published:** 2017-08-18

**Authors:** Min Cui, Bo Jia, Huanhuan Liu, Xin Kan, Yu Zhang, Ronghua Zhou, Zhipeng Li, Liang Yang, Dexiang Deng, Zhitong Yin

**Affiliations:** ^1^Jiangsu Key Laboratory of Crop Genetics and Physiology, Co-Innovation Center for Modern Production Technology of Grain Crops, Key Laboratory of Plant Functional Genomics of the Ministry of Education, Yangzhou University Yangzhou, China; ^2^Huaiyin Institute of Agricultural Sciences of Xuhuai Region in Jiangsu Huai’an, China

**Keywords:** flowering time, leaf number above the primary ear, maize, plant height, quantitative trait loci (QTL)

## Abstract

The leaf number above the primary ear (LA) is a major contributing factor to plant architecture in maize. The yield of leafy maize, which has extra LA compared to normal maize, is higher than normal maize in some regions. One major concern is that increasing LA may be accompanied by increased plant height and/or flowering time. Using an F_2:3_ population comprising 192 families derived from a leafy maize line and a normal maize line, an association population comprising 437 inbred maize lines, and a pair of near-isogenic maize lines, we mapped the quantitative trait loci (QTL) associated with LA and assessed its genetic relationship with flowering time and plant height. Ten QTL with an additive and dominant effect, 18 pairs of interacting QTL in the F_2:3_ population and seventeen significant SNPs in the association population were detected for LA. Two major QTL, *qLA3-4* and *qLA7-1*, were repeatedly detected and explained a large proportion of the phenotypic variation. The *qLA3-4* was centered on *lfy1*, which is a dominant gene underlying extra leaves above the ear in leafy maize. Four LA QTL were found to overlap with flowering time and/or plant height, which suggested that these QTL might have a pleiotropic effect. The pleiotropy of the *lfy1* locus on LA, flowering time and plant height were validated by near-isogenic line analysis. These results enhance our understanding of the genetic architecture affecting maize LA and the development of maize hybrids with increased LA.

## Introduction

The leaf number above the primary ear (LA) is an essential canopy architecture trait for maize. The photosynthate used for grain filling mainly comes from leaves at and above the primary ear (Shaver, 1983). During grain filling, the leaves above the primary ear intercept radiation better than those below the primary ear in maize plants (Tollenaar and Dwyer, 1997), and they are also younger and more metabolically active (Tollenaar and Dwyer, 1997). Plants with more LA have potentially greater photosynthetic potential due to enhanced source strength, which permits greater accumulation and transport of dry matter into the grain (Shaver, 1983; Na et al., 2006). An increase of LA can increase the leaf area and plant photosynthate production during grain filling (Tollenaar and Dwyer, 1997; Andrews et al., 2000). Furthermore, an increase of LA can lower the ear placement and improve plant standability (Muirhead and Shaver, 1985). However, an increase of LA may increase water demand due to the increased leaf area, which is an undesirable trait in some water-limited environments (Lambert et al., 2014).

Variation in LA is generally limited in common maize and ranges from four to seven. An exception is observed in leafy maize, which has extra LA (Shaver, 1983). The leafy maize has 7–25 leaves above the primary ear and a similar number of leaves under the primary ear compared to common maize (Shaver, 1983; Chen et al., 2007). This leafy trait has been used in maize hybrid breeding. Several silage maize hybrids with extra LA have been registered in Hungary and are on the European Union’s Variety List (Chen et al., 2007). Furthermore, to adapt to short-season environments such as Canada, the reduced stature (*rd1*) trait was introduced to reduce the flowering time of leafy maize (Modarres et al., 1997). Hybrids bearing reduced stature and leafy traits were shown to exhibit higher yield and higher population-density tolerance and were less affected by weed pressure than conventional maize hybrids (Begna et al., 2001). In a few cases, some leafy maize hybrids with higher photosynthetic sources did not show increased grain yield (Dwyer et al., 1995). This might be due to a weaker translocation of the photosynthate within plants and/or a smaller sink.

Although LA is an important trait, the genetic mechanisms underlying this trait are largely unknown. Few studies reported mapping quantitative trait loci (QTL) for LA in maize. In a common maize BC_2_S_3_ population, 15 QTL for LA were detected, and one major-effect QTL was delimited to a 20 Mb genomic region using a fine mapping method (Li D. et al., 2016). In a leafy maize derived F_2_ population, a major locus underlying the leafy trait was first mapped to the long arm of chromosome 3 (Oishi et al., 2009). In a recent study, this locus was further mapped to a 55 kb genomic interval (Du et al., 2015). In a breeding program, only the QTL, which can be expressed in different genetic backgrounds and/or under multiple environments, are highly valuable. Therefore, further LA QTL mapping in a wide range of maize materials under multiple environments is needed.

One important factor to consider in developing maize hybrids with increased LA is the flowering time. Flowering time is a critical factor for maize adaptation to local environments. An increase of maize LA might be accompanied by a longer flowering time and thus prevent its use in short growing-season environments. Leafy maize with additional LA is indeed classified as late flowering (Neuffer et al., 1997). Recently, the genetic relationship between LA and the flowering time has been dissected using a BC_2_S_3_ population (Li D. et al., 2016). The results showed that the flowering time was mainly affected by the leaf number below the primary ear rather than LA. However, the genetic relationship between leafy traits and flowering time is not yet known.

Another consideration when utilizing maize hybrids with increased LA is plant height. Greater LA is thought to be associated with increased plant height in maize. Taller maize hybrids are more susceptible to lodging, and this decreases their potential adaptability to a high population density, which is key to achieving a high grain yield in modern maize production (Cai et al., 2012). This phenotypic relationship also occurs in species of rice and wheat. During the so-called “green revolution,” semi-dwarf rice and wheat varieties were widely used due to their increased tolerance to high population densities and resistance to lodging. These varieties therefore achieved higher grain yields (Waines and Ehdaie, 2007). To date, many QTL for plant height have been detected using different linkage mapping populations and/or diverse association panels (Wang and Li, 2008; Peiffer et al., 2014; Li X. et al., 2016), and several genes related to these QTL have been successfully cloned (Teng et al., 2013; Xing et al., 2015). However, little information exists regarding the genetic relationship of these QTL or genes to QTL for LA in maize, especially at the population level. Further genetic studies are needed to investigate the level of genetic sharing between LA and plant height in maize.

In the present study, QTL for LA were mapped in 192 F_2:3_ families derived from the leafy maize line Y915 and the normal maize line Z58 in three field-grown environments and in a diverse association panel comprising 437 normal inbred lines in two field-grown environments. In addition, to investigate the genetic relationship of LA to plant height and flowering time, the QTL for these two traits were mapped in the F_2:3_ families. Finally, one pair of near-isogenic lines was employed to confirm the effects of the leafy gene on the plant height and flowering time.

## Materials and Methods

### Plant Materials, Plant Growth Conditions, and Phenotypic Evaluation

The F_2:3_ bi-parental population, which was developed from a cross between the leafy maize inbred line Y915 and the normal line Z58, was used to map QTL. The inbred line Y915 carrying the *lfy*1 gene was developed from a leafy hybrid introduced from America. Z58 is the female parent of the commercial hybrid ZhengDan958, which is currently grown in China. The F_2:3_ population contained 192 F_2:3_ families developed by self-pollinating 192 F_2_ individuals.

For the genome wide association studies (GWAS) experiment, 437 maize lines were chosen from a previously described association population (Yang et al., 2011). These chosen lines exhibited normal growth and maturation in Yangzhou.

Two near-isogenic maize lines (NILs), NIL^6L^ and NIL^9L^, were developed from a cross between Y915 and a normal line Y53. Y53 is the male parent of the commercial hybrid Suyu 16. The details on how the NILs were developed is shown in **Supplementary Figure S1**. Briefly, the F_1_ plants of this cross were self-pollinated for six generations in field-grown conditions. In each generation, plants showing the leafy trait and segregation for LA in their progenies were selected for continuously self-pollinating in the field. After six generations of self-pollination, two F_8_ homozygous inbred lines were developed from two F_7_ individuals of a single F_6_ plant. The two homozygous inbred lines stably express 6 and 9 LA, respectively.

The F_2:3_ population was planted in the following three locations: (1) The Experimental Farm of the Agricultural College of Yangzhou University (N:32.40°, E:119.40°), where seeds were sown on March 31st, 2016. The mean day lengths were greater than 13 h during the growing season; (2) the Experimental Farm of Jiangsu Huai’an Institute of Agricultural Sciences (N:33.62°, E:119.02°), where seeds were sown on June 19th, 2016. The mean day lengths were greater than 13 h during the growing season; and (3) the winter nursery of Hainan Province (N:18.73°, E:109.17°), where the seeds were sown on November 30th, 2016. The mean day lengths were less than 13 h during the growing season. All of the F_2:3_ lines were grown in a randomized complete-block design with two replications in each location. The association population and the two NILs were planted in two locations: (1) The Agricultural College of Yangzhou University, where seeds were sown on March 31st, 2016 and (2) the winter nursery of Hainan Province, where the association population seeds were sown on October 15th, 2015, and the NILs seeds were sown on November 30th, 2016. The association population of 437 lines and the two NILs were also planted in a randomized complete-block design in each growing location. The 437 lines were planted with three replicates, and the two NILs were planted in two replicates. For each accession, 10 plants were planted in 2.9 m row plots with 0.55 m row spacing. The field management was performed according to the standard agronomic practices in each location. In each plot, seven consecutive plants were selected for the measurements of LA, plant height and days to tasseling. The phenotype of each line was obtained by averaging the phenotypic values of the seven measured plants.

### Linkage Map Construction

Randomly selected 192 F_2_ individuals derived from the leafy maize inbred line Y915 and the normal line Z58 were used to construct a genetic linkage map. Leaf genomic DNA samples were prepared using the CTAB method (Murray and Thompson, 1980). All of the F_2_ individuals and their parent lines were genotyped using a single nucleotide polymorphism (SNP) chip. The SNP chip included 56,110 random SNP markers that evenly cover the maize genome. The SNP genotyping was conducted using the GoldenGate assay at the China Golden Marker Co. Ltd. Linkage analysis was performed using the JoinMap version 4.0 (Stam, 1993). A likelihood of odds (LOD) threshold of 3.0 was used to infer the linkage. The maximum distance between two loci used for determining the linkage groups was set as 50 cM. The Kosambi function was used to calculate the map distances (cM) (Kosambi, 1943). All linkage groups were assigned to particular chromosome, and the F_2_ linkage maps were oriented based on the physical position of SNP markers (Boopathi, 2013).

### Statistical Analysis and QTL Mapping

Descriptive statistical analyses, frequency distribution analyses, analysis of variance (ANOVA), and correlation analyses were performed using SPSS Statistics 17.0 software (SPSS Inc., Chicago, IL, United States). The broad-sense heritability was calculated according to our previous method (Yin et al., 2014).

Two QTL mapping methods were used. First, a composite interval mapping (CIM) method was used to map QTL with additive and dominant effects separately in an individual environment using Win-QTLCart v2.5 software (Wang et al., 2012). Second, the ICIM-EPI method was employed to map QTL with epistatic effects using QTL IciMapping v4.0 software (Meng et al., 2015). The POUT, PIN and scanning steps were set at 0.0002, 0.0001, and 5 cM, respectively. The LOD thresholds for declaring a significant QTL were determined by 1000 permutation tests at the 95% confidence level. If two QTL have an overlapped confidence interval, they were considered common QTL. To investigate whether two QTL from different studies were common QTL, the confidence interval of each QTL was obtained using the physical location of two SNP markers that flanked the QTL confidence interval. The physical position of the SNP markers was inferred by blasting against the maize B73 line genomic sequence (B73 RefGen_v2). QTL with overlapping intervals on the maize B73 genomic sequence were considered common QTL.

Genome wide association studies was performed using TASSEL 5.0 software (Bradbury et al., 2007). The population structure (Q) and the kinship matrix (K) was included in the statistical model to reduce spurious associations (Yang et al., 2011). The Q was calculated using Structure 2.3 software. The K was calculated using Powermarker 3.25 software. Three statistical models were evaluated in the GWAS experiments as the following: (1) a GLM model without Q and K; (2) a GLM model with Q. Q serves as a cofactor to correct for the population structure; and (3) an MLM model with Q and K. Q and K serve as cofactors to correct for the population structure. According to the quantile-quantile (Q-Q) plot, the MLM model incorporating Q and K was suitable for this study. Markers were identified as significantly associated with traits via comparisons using the Bonferroni threshold (Zhang et al., 2009).

### QTL Correspondence between Different Traits

The random chance of obtaining the observed number of overlapped QTL between two compared traits (*p*) was calculated using the following equation:

$$p=\frac{\left(\begin{array}{c} \begin{array}{c} l\\ m \end{array} \end{array})(\begin{array}{c} \begin{array}{c} n-1\\ s-m \end{array} \end{array}\right)}{\left(\begin{array}{c} \begin{array}{c} n\\ s \end{array} \end{array}\right)}$$

(Lin et al., 1995; Feltus et al., 2006; Li D. et al., 2016). The *n* refers to the total number of comparison intervals. In this study, *n* was 115, which was obtained by dividing the total length of the linkage map by the average interval of QTL. The total length of the linkage map of the F_2:3_ population was 1608 cM. The average interval of the QTL was 14 cM. For the two compared traits, *l* refers to the larger number of QTL detected for them, *s* refers to the smaller number of QTL, and *m* refers to the number of overlapped QTL.

## Results

### Genetic Linkage Map

An SNP chip returning 56110 markers was used to genotype the leafy maize line Y915, the normal line Z58, and the F_2_ individuals derived from these two parent lines. Of the 56,110 SNPs, 14,283 were polymorphic in both the parents and the F_2_ individuals. According to a haplotype analysis using Haploview 4.2 (Barrett et al., 2005), some of these polymorphic markers co-segregated. Eventually, 1,736 SNP markers representing recombination bins were selected to construct the linkage map (**Supplementary Figure S2**). The markers were uniformly distributed along the chromosomes. The whole map spanned 1,608 cM on 10 linkage groups, and there was an average distance of 0.93 cM between two neighboring markers. Chromosome 1 contained the largest number of markers (up to 270), while chromosome 10 had only 80 markers.

### Phenotypic Analysis

**FIGURE 1:**
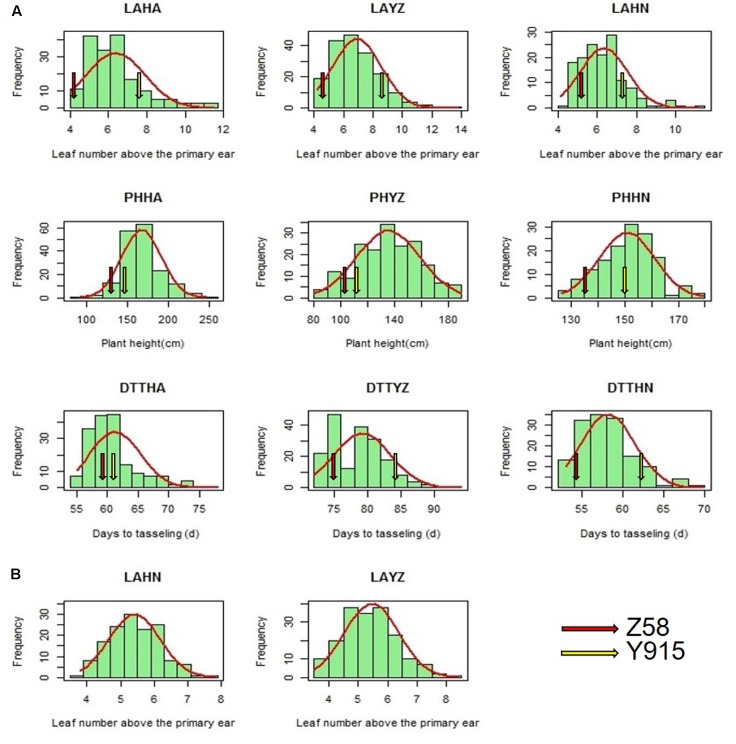
The frequency distribution of the leaf number above the primary ear (LA), plant height (PH), and the days to tasseling (DTT). **(A)** Frequency distribution in the F_2:3_ population. The red and yellow arrows represent Z58 and Y915 in different environments, respectively. **(B)** Frequency distribution in the association population. HA, YZ, and HN represent Huai’an, Yangzhou and Hainan, respectively.

The distribution of LA, plant height, and days to tasseling in the F_2:3_ population and LA in the association population were continuous and relatively normal (**Figures 1A,B**). The means, standard deviations, ranges, skewness, broad-sense heritability values, and ANOVA for LA, plant height, and days to tasseling are presented in **Table 1**. The LA in the two populations and the plant height and days to tasseling in the F_2:3_ population displayed a wide range. Compared with Z58, the Y915 line had a significantly greater LA, higher plant height, and more days to tasseling, and the transgressive segregation of these traits was apparent in the F_2:3_ population. The phenotypic value of LA of the combined environments ranged from 4 to 14 in the F_2:3_ population and from 2.5 to 8.5 in the association population. The plant height values of combined environments in the F_2:3_ population ranged from 70.33 to 245.86 cm. The days to tasseling values of the combined environments in the F_2:3_ population ranged from 52 to 98 days.

**Table 1:** Descriptive statistics, ANOVA and broad-sense heritability for leaf number above the primary ear (LA), plant height (PH), and days to tasseling (DTT).

Traits	Environment	Z58 Mean ± *SD*	Y915 Mean ± *SD*	Mean ± *SD*	Range	Skew	G	G^∗^E	*H^2^*
LA (F_2:3_)	2016 Yangzhou	4.75 ± 0.50	8.67 ± 0.58	6.88 ± 1.71	4.00 – 13.00	0.127	4.987^∗∗^		85%
	2016 Huai’an	4.25 ± 0.50	7.80 ± 0.44	6.28 ± 1.52	4.00 – 11.50	0.126	4.253^∗∗^		92%
	2016 Hainan	5.21 ± 0.42	7.61 ± 0.86	6.33 ± 1.33	4.00 – 14.00	0.135	2.955^∗∗^		83%
	Combined	4.77 ± 0.44	7.93 ± 0.61	6.58 ± 1.64	4.00 – 14.00	0.090	8.195^∗∗^	1.09^∗∗^	83%

PH (cm) (F_2:3_)	2016 Yangzhou	104.40 ± 7.12	108.33 ± 4.72	133.81 ± 23.52	70.33 – 199.20	0.129	759.01^∗∗^		69%
	2016 Huai’an	136.38 ± 14.80	149.72 ± 12.02	166.69 ± 23.34	90.50 – 245.86	0.129	923.62^∗∗^		85%
	2016 Hainan	136.08 ± 12.39	148.42 ± 11.23	151.51 ± 13.40	113.00 – 188.00	0.135	229.80^∗∗^		63%
	Combined	127.50 ± 19.59	140.86 ± 20.62	150.24 ± 28.62	70.33 – 245.86	0.091	1370.98^∗∗^	311.65^∗∗^	74%

DTT (d) (F_2:3_)	2016 Yangzhou	74.33 ± 0.58	85.00 ± 1.41	79.03 ± 4.56	70.00 – 98.00	0.129	33.30^∗∗^		80%
	2016 Huai’an	59.00 ± 1.41	60.00 ± 1.41	61.18 ± 3.96	56.50 – 78.00	0.182	31.34^∗∗^		91%
	2016 Hainan	53.33 ± 1.53	63.33 ± 1.15	58.02 ± 3.51	52.00 – 69.00	0.135	18.61^∗∗^		76%
	Combined	68.20 ± 8.43	75.20 ± 13.91	70.11 ± 9.93	52.00 – 98.00	0.092	55.07^∗∗^	9.57^∗∗^	78%

LA (Association	2015 Hainan			5.25 ± 0.78	2.50 – 7.80	0.091	1.093^∗∗^		90%
population)	2016 Yangzhou			5.33 ± 0.83	3.33 – 8.50	0.092	1.249^∗∗^		90%
	Combined			5.29 ± 0.81	2.50 – 8.50	0.065	1.173^∗∗^	2.16^∗∗^	90%


*^∗∗^Significant at *p < 0.01*; NS, not significant; G, Genotype; G^∗^E, Genotype × Environment; H^*2*^, Broad-sense heritability.*

The genotypic variance and the genotypic-by-environment variance of the three traits were significant in the F_2:3_ and association populations. The heritability (*H^2^*) estimates in the individual environment ranged from 83 to 92% for LA, from 63 to 85% for plant height, and from 76 to 91% for days to tasseling. Overall, the maize plants clearly showed considerable natural variation in LA, plant height, and days to tasseling and displayed an abundant genetic diversity.

### QTL Analysis for LA

#### QTL Mapping for LA Using Linkage Analysis

**FIGURE 2:**
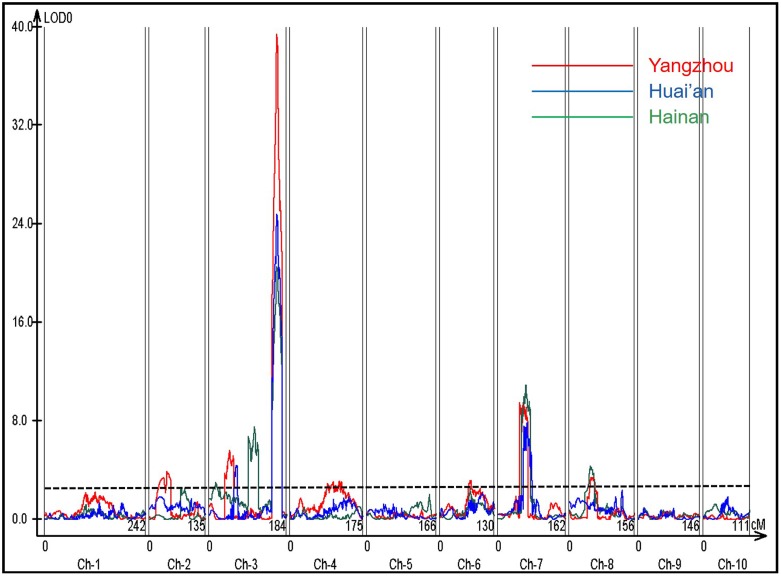
Quantitative trait loci (QTL) for LA mapped in the F_2:3_ population. The *x*-axis shows the genetic position along the chromosomes. A vertical bar separates adjacent chromosomes. The *y*-axis represents the logarithm of the odds (LOD) score of each scanning position. The dotted line represents the LOD significance threshold. Red, blue and green represent Yangzhou, Huai’an and Hainan, respectively.

Quantitative trait loci for LA was identified in the F_2:3_ population separately in each environment. Ten QTL were detected across four chromosomes (**Table 2** and **Figure 2**); *qLA3-4* on chromosome 3 and *qLA7-1* on chromosome 7 were detected across all of the environments. *qLA3-4* explained the largest phenotypic variance and ranged from 38 to 59% in the three environments. A survey on the B73 maize inbred line reference genome (B73 RefGen_v2) showed that the genomic interval containing the *lfy1* gene (Du et al., 2015) is under the peak of *qLA3-4. qLA7-1* explained the second largest phenotypic variance and ranged from 10 to 20% in the three environments. Additive effect values indicated that Y915 alleles increased LA at *qLA3-4*, whereas Z58 alleles increased LA at *qLA7-1* (**Table 2**). Dominance effects showed that *qLA3-4* displayed positive dominance effects, whereas *qLA7-1* showed negative dominance effects. The remaining QTL were detected in one or two environments. The phenotypic variances explained by these QTL were small and mostly less than 5%.

**Table 2:** Quantitative trait loci (QTL) for LA detected in different environments.

Trait	QTL	Chr	Marker interval	Position (cM)	Yangzhou				Huai’an				Hainan			
							
					LOD	Add^a^	Dom^b^	*R^2^*^c^ (%)	LOD	Add	Dom	*R^2^* (%)	LOD	Add	Dom	*R^2^* (%)
LA	*qLA 2-1*	2	SYN28948–PZE-102062967	33.4–37.1	3.43	-0.36	-0.28	0.9								
	*qLA 2-2*	2	SYN18801–PZE-102084025	40.3–52.5	3.93	-0.40	-0.26	1.2								
	*qLA 3-1*	3	SYN22785–PZE-103013846	14.5–22.8					3.00	0.37	-0.29	5.5				
	*qLA 3-2*	3	PZE-103019119–PZE-103062384	35.6–64.5	5.21	0.50	0.07	3.8					4.41	0.44	0.01	6.1
	*qLA 3-3*	3	PZE-103102047–PZE-103147317	91.4–114.1					7.55	0.69	0.33	4.6				
	*qLA 3-4*	3	SYN32259–PZE-103182430	148.9–173.2	39.47	-1.87	0.09	58.9	20.57	-1.38	0.14	38.2	24.81	-1.29	0.05	52.1
	*qLA 4-1*	4	PZE-104091188–PZE-104114030	88.4–122.2	2.92	-0.34	-0.15	1.3								
	*qLA 6-1*	6	SYN31423–PZE-106050426	71.4–96.1	3.17	0.24	0.35	1.0					2.55	0.14	0.19	1.3
	*qLA 7-1*	7	PZE-107025581–SYN21763	51.1–71.2	9.26	0.71	-0.12	10.3	10.01	0.82	-0.40	20.3	7.58	0.52	-0.40	15.9
	*qLA 8-1*	8	SYN8904–PZE-108090276	48.7–61.9	3.44	-0.42	0.10	3.2	3.78	-0.57	0.34	7.3				


*^a^Positive values indicate increasing effects contributed by Z58, and negative values indicate increasing effects contributed by Y915 in the F_*2:3*_ population. ^b^Positive values of the dominance effect indicate that the heterozygotes have higher phenotypic values than the respective means of two homozygotes. Negative values indicate that heterozygotes have lower phenotypic values than the respective means of two homozygotes. ^c^Percentage phenotypic variation explained by QTL.*

**FIGURE 3:**
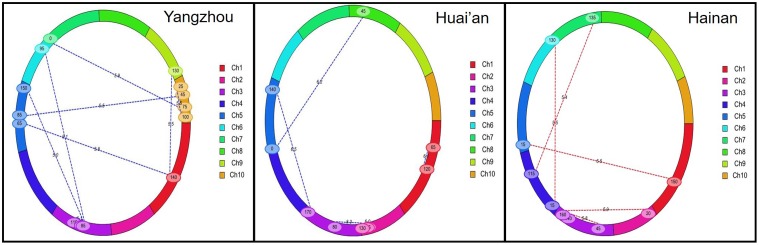
Epistatic effects on the leaf number above the primary ear (LA) in different environments. The lines denote epistatic associations between QTL.

To examine the epistatic interactions, eighteen pairs of interacting QTL for LA were mapped to chromosomes 1, 2, 3, 4, 5, 7, and 10 (**Table 3** and **Figure 3**). These interactions explained the phenotypic variation ranging from 2.7 to 12.2%. No pairs of interacting QTL were stably detected across the different environments. Except for the interaction between QTL on chromosomes 2 and 3 in Huai’an, no pairs of interacting QTL could explain more than 10% of the measured phenotypic variation. Of the two LA QTL detected with a large effect, *qLA3-4* showed a significant epistatic interaction with QTL on chromosomes 2 and 5 in Huai’an, whereas *qLA7-1* did not show a significant epistatic interaction.

**Table 3:** Epistatic loci for LA in different environments.

Environments	Chr.	Left marker	Right marker	Chr.	Left marker	Right marker	LOD	*R^2^* (%)	Add × Add
Yangzhou	1	PZE-101154812	SYN28787	9	PZE-109115895	PZE-109119211	5.45	3.2	0.47
Yangzhou	1	PZE-101154812	SYN28787	5	PZE-105053187	PZE-105053583	5.79	2.7	0.75
Yangzhou	3	PZE-103100051	SYN19496	5	PZE-105175232	PZE-105177702	5.02	3.0	-0.50
Yangzhou	3	PZE-103094435	PZE-103096203	6	PZE-106038186	SYN21273	5.07	3.1	-0.01
Yangzhou	3	PZE-103100051	SYN19496	3	PZE-103132112	PZE-103132539	6.13	3.1	-0.65
Yangzhou	5	PZE-105101487	PZE-105102393	10	PZE-110054937	PZE-110055126	5.48	2.7	-0.47
Yangzhou	7	PZA03504.1	SYN10729	10	SYN13121	PZE-110094849	5.88	2.7	0.38
Yangzhou	10	SYN4503	PUT-163a-6022278-1333	10	PZE-110102725	PZE-110108464	5.52	2.9	-0.41

Huai’an	1	SYN6094	PZE-101053346	1	SYN2723	SYN38676	6.11	5.9	-1.54
Huai’an	2	SYN31053	PZE-102175138	2	SYN12385	PZE-102177164	6.01	6.8	0.27
Huai’an	2	SYN15515	PZE-102181729	3	PZE-103087716	PZE-103088747	8.29	12.2	0.47
Huai’an	3	SYN8581	PZE-103182430	5	PZE-105160758	SYN22486	6.51	7.7	0.36
Huai’an	5	PZE-105000103	SYN9877	8	PUT-163a-60398609-1977	SYN18175	6.19	7.6	0.70

Hainan	1	PZE-101160778	PZE-101161325	5	SYN35358	SYN31758	5.48	5.0	-0.66
Hainan	2	PZE-102027614	SYN28948	3	SYN38131	PZE-103179063	5.86	4.9	-0.27
Hainan	3	PZE-103028926	PZE-103029945	3	PZE-103056912	PZE-103165394	5.83	4.9	-0.37
Hainan	4	ZM013591-0465	SYN36763	6	SYN11457	PZE-106006362	5.47	5.1	-0.17
Hainan	4	PZE-104107791	PZE-104109358	7	PZE-107128836	PZE-107130734	5.41	4.8	-0.47


*Add × Add indicates an epistatic effect for the QTL; *R^*2*^* percentage phenotypic variation explained by QTL. All estimated values were significant at a probability level of 0.005.*

#### Association Mapping for LA

**FIGURE 4:**
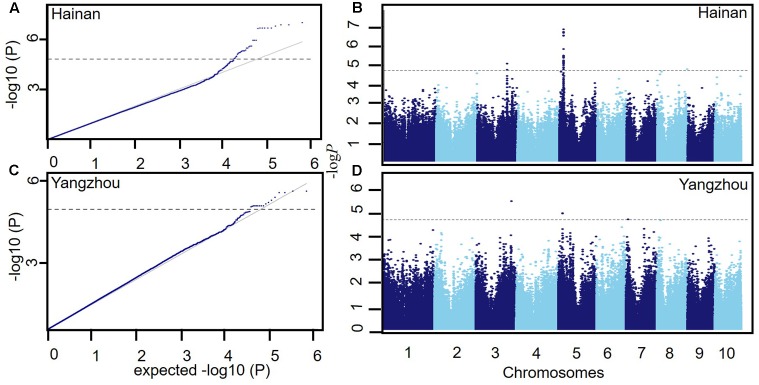
The genome-wide association analysis for LA in the different environments. **(A,C)** Q-Q plots for the LA trait in Hainan and Yangzhou. Q-Q plots for the marker-trait association analysis for LA were generated using the MLM + Q + K method. The gray line is the expected line under a null distribution. **(B,D)** The Manhattan plots for the LA trait in Hainan and Yangzhou. The dashed line indicates a significant association signal.

We performed GWAS for LA with 558,629 SNP markers and the phenotypic LA values obtained in an association population including 437 maize lines to map the LA loci. The population structure can lead to spurious associations between the markers and phenotypes in the GWAS analyses. As shown in the Q–Q plots (**Figures 4A,C**), the effect of the population structure on LA was reduced by using the MLM model with Q and K, and the *P*-values from this model are nearly equal to the expected values.

Seventeen SNP markers for LA were identified as having significant marker-trait associations at the Bonferroni-adjusted significance threshold (**Table 4** and **Figures 4B,D**). The significant SNP markers were located on chromosomes 3, 5, 7, and 8 and explained 4.5–6.8% of the phenotypic variation. Of the seventeen significant marker-trait associations, thirteen and four were detected in Hainan and Yangzhou, respectively. Further analysis showed that the ten significant SNP markers on chromosome 5 were located in two linkage disequilibrium (LD) blocks (**Supplementary Figure S3**). Notably, one of the highly significant SNP markers, Chr5.S_24437183, was repeatedly detected for LA in the two environments. In addition, two significant SNPs detected on chromosome 3, Chr3.S_173016406 and Chr3.S_173016373, fell within the *qLA3-3* region detected for LA in the F_2:3_ population.

**Table 4:** Single nucleotide polymorphism (SNP) markers associated with LA in 437 inbred lines in different environments.

Environments	Traits	Chr.	Marker position (Mb)	*P^a^*	-log*P*	*R^2b^ (%)*
Hainan	LA	3	Chr3.S_173016406	8.20E-06	5.08	4.9
	LA	3	Chr3.S_173016373	1.69E-05	4.77	4.5
	LA	5	Chr5.S_24417652	1.35E-07	6.86	6.8
	LA	5	Chr5.S_24418518	1.81E-07	6.74	6.6
	LA	5	Chr5.S_24418524	1.81E-07	6.74	6.6
	LA	5	Chr5.S_24417274	2.11E-07	6.67	6.6
	LA	5	Chr5.S_24417386	2.78E-07	6.55	6.4
	LA	5	Chr5.S_24417411	2.78E-07	6.55	6.4
	LA	5	Chr5.S_24417416	2.78E-07	6.55	6.4
	LA	5	Chr5.S_24417422	2.78E-07	6.55	6.4
	LA	5	Chr5.S_24417487	2.78E-07	6.55	6.4
	LA	5	Chr5.S_24437183	2.94E-07	6.53	6.8
	LA	8	PZE-108127945	1.59E-05	4.80	4.9

Yangzhou	LA	3	PZE-103149689	2.95E-06	5.53	6.4
	LA	5	Chr5.S_24436668	9.62E-06	5.02	5.6
	LA	5	Chr5.S_24437183	9.91E-06	5.00	5.8
	LA	7	PZE-107015746	1.78E-05	4.76	6.3


*^a^*P*-value from ANOVA analysis of the mean LA from three replicates; ^b^*R^*2*^* percentage of phenotypic variation explained by ANOVA.*

### Genetic Relationship of LA to Flowering Time and Plant Height

**FIGURE 5:**
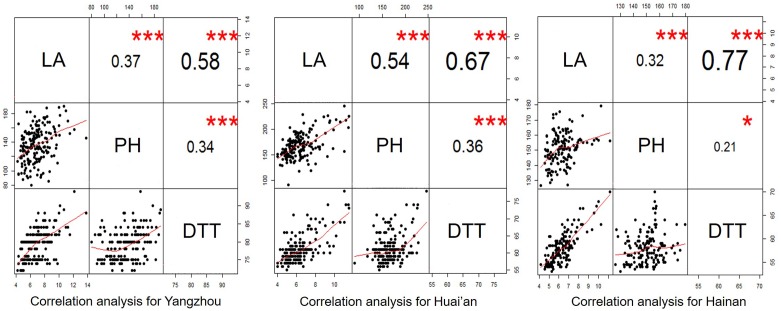
Correlation analysis for the leaf number above the primary ear (LA), plant height (PH) and the days to tasseling (DTT) in the F_2:3_ population. The values above the diagonal are pairwise correlation coefficients between traits, and the plots below the diagonal are scatter plots of the compared traits. ^∗∗∗^ significant at *p* < 0.001; ^∗^ significant at *p* < 0.05.

Correlation analysis among LA, plant height, and days to tasseling was performed, and the results are presented in **Figure 5**. The correlations were strikingly similar in different environments. The three traits showed a positive relationship with each other. All phenotypic correlations reached significance at *p* < 0.001 or *p* < 0.05, but the correlation coefficient varied substantially between the traits. The phenotypic correlation between LA and days to tasseling was higher than that between LA and plant height.

To investigate the genetic relationship between LA and days to tasseling and plant height, the QTL for days to tasseling and plant height was also mapped in the F_2:3_ population and compared with those identified for LA in the same population as described above. Information for the plant height and days to tasseling QTL is listed in Supplementary Table S1. Ten QTL for days to tasseling were identified on chromosomes 1, 3, 5, 6, and 7. These QTL explained the phenotypic variation ranging from 2.2 to 29.9% with LOD scores ranging from 2.57 to 25.57. Eleven QTL for plant height were identified on chromosomes 1, 3, 5, 6, 7, and 9. These QTL explained phenotypic variation ranging from 0.9 to 19.9% with LOD scores ranging from 2.50 to 8.15.

**FIGURE 6:**
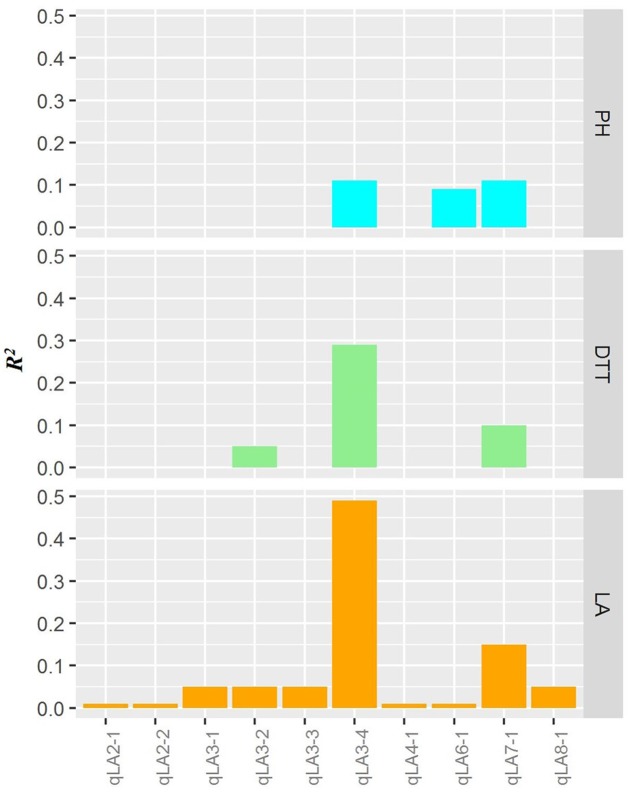
Average phenotypic variation explained by LA QTL in the F_2:3_ population. The *x*-axis indicates the QTL for LA. The *y*-axis indicates the average phenotypic variation explained by the QTL for each trait. LA, the leaf number above the primary ear; PH, plant height; DTT, days to tasseling.

The LA QTL *qLA3-4*, *qLA7-1* and *qLA3-2* were found to have significant effects on the days to tasseling (**Figure 6**, **Table 2** and Supplementary Table S1), and these QTL explained 28.5, 9.8, and 5.5%, respectively, of the phenotypic variation of average days to tasseling. Similarly, the LA QTL *qLA3-4*, *qLA7-1* and *qLA6-1* were found to have significant effects on plant height, and these QTL explained 10.2, 11.2, and 8.5%, respectively, of the phenotypic variation of the average plant height. Notably, the two QTL, *qLA3-4* and *qLA7-1*, exhibited pleiotropic effects for both days to tasseling and plant height. All coincident QTL showed additive effects in the same direction for the respective traits (**Table 2** and Supplementary Table S1). The chance of the occurrence of these coincident QTL among LA, days to tasseling, and plant height is significantly lower than by chance alone (Supplementary Table S2).

### Validation of *qLA3-4* in NILs

**FIGURE 7:**
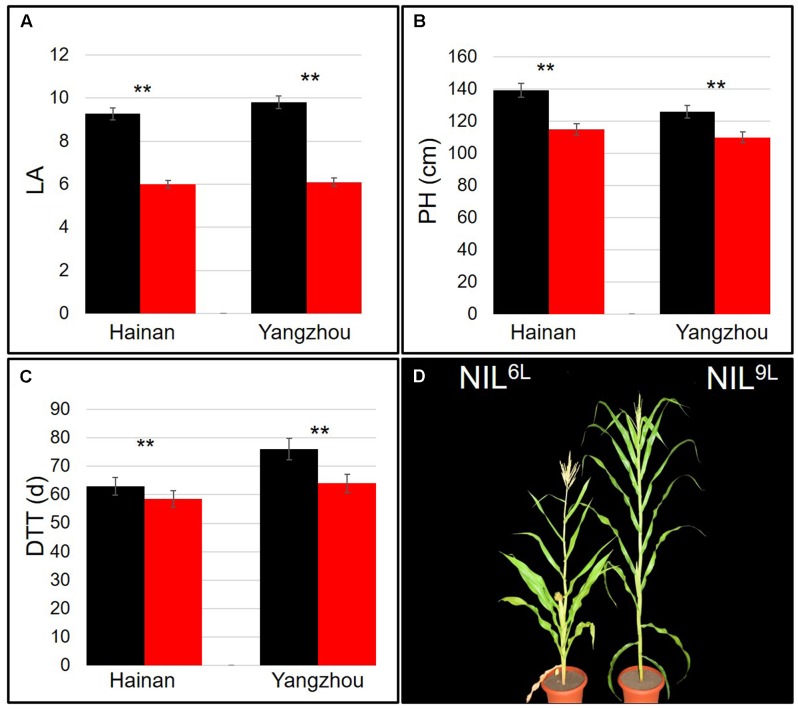
Phenotypes and phenotypic effect analysis of near-isogenic lines (NILs) for *qLA3-4*. **(A–C)** The black and red bars represent NIL^9L^ and NIL^6L^ in different environments, respectively. The phenotypic values are shown as the mean ± SE; ^∗∗^ significant at *P* < 0.01; LA, leaf number above the primary ear; PH, plant height; and DTT, days to tasseling. **(D)** The phenotypes of NIL.

Two near-isogenic maize lines (NILs) with different LA were developed from a single F_6_ individual arising from a cross between leafy maize line Y915 and the normal line Y53. The two NILs, designated NIL^6L^ and NIL^9L^, have six and nine LA, respectively (**Figures 7A,D**). We genotyped the two NILs with 518 SSR markers, which were obtained from the Mazie Database website (Sen et al., 2009). Fifteen markers were chosen to cover the *qLA3-4* region, and the remaining markers were chosen to generate an even distribution along the maize ten chromosomes except for the *qLA3-4* region. Of the 15 markers that covered the *qLA3-4* region, 13 SSR markers fell between the two SNP markers that flanked the *qLA3-4* confidence interval, and two SSR markers covered the two flanking SNP markers. Only 10 SSR markers were observed to be polymorphic between the two lines (**Supplementary Figure S4**). These polymorphic markers covered a genomic region of approximately 8.3-Mb in the maize line B73 reference genome. These findings suggested that the *qLA3-4* region could underlie the observed LA difference in two NILs, which agree with the QTL mapping results in the F_2:3_ population.

To validate the genetic effect of *qLA3-4* on plant height and the days to tasseling observed in the F_2:3_ population, NIL^6L^ and NIL^9L^ were investigated for plant height and days to tasseling. Significant phenotypic differences in plant height and days to tasseling were found to exist in the two NILs (**Figures 7B,C**). These results further suggest that *qLA3-4* affects not only LA but also plant height and days to tasseling.

## Discussion

In the present study, LA exhibited a relatively normal distribution in the leafy maize derived F_2:3_ population and in the association population (**Table 1** and **Figure 1**). This finding suggests that LA is a quantitative trait. However, in a previous study by Du et al. (2015), the distribution of LA was shown to be controlled by a single dominant gene in an F_2_-segregating population derived from a normal line (B73) and a leafy maize line (CO412). This inconsistency suggests that LA is largely dependent on the genetic background of the population under study (Colasanti and Muszynski, 2009). In practice, LA has been found to exhibit different distributions in different leafy maize derived populations and has either been considered a quantitative trait or a qualitative trait (Oishi et al., 2009; Du et al., 2015). Since flowering time and plant height were observed to have a close relationship with LA (**Figure 5**), the different distribution of LA in the different genetic population might likely depend on what flowering time and plant height genes are segregating in the population. If the flowering time and plant height genes are fixed, LA might appear to be a qualitative trait, but when major genes controlling height and flowering are segregating, LA might appear to be a quantitative trait.

In the F_2:3_ population, the linkage mapping analysis detected 10 additive and dominant QTL and eighteen pairs of interacting QTL for LA (**Tables 2**, **3** and **Figures 2**, **3**). These results suggest that in addition to QTL with additive and dominant effects, epistatic QTL effects also have contributions on LA in maize. However, compared to the additive and dominant QTL, the interacting QTL explained a much smaller proportion of phenotypic variation, especially in Yangzhou and Hainan (**Tables 2**, **3**), which suggests that additive and dominant QTL played a more important role in determining LA. Of the ten additive and dominant QTL, two QTL (*qLA3-4* on chromosome 3 and *qLA7-1* on chromosome 7) were repeatedly detected across all three environments, and they explained a large proportion of the phenotypic variation. In the association population, GWAS revealed seventeen significant SNPs for LA (**Table 4** and **Figure 4**). Much fewer SNP markers were detected in Yangzhou (4 SNPs) than in Hainan (13 SNPs). This might be caused by the environmental effects. The locus containing SNP Chr5.S_24437183 was repeatedly detected in different environments. In addition, two significant SNPs detected on chromosome 3, Chr3.S_173016406 and Chr3.S_173016373 fell within the *qLA3-3* region detected for LA in the F_2:3_ population. These stable and consistent loci for LA should be considered priority candidates for MAS.

A dominant gene conferring extra LA in maize, named *lfy1*, was previously mapped to a 55 kb interval on chromosome 3 using an F_2_-segregating population derived from leafy maize line (CO412) and normal line (B73) (Du et al., 2015). In the present study, *qLA3-4* was identified as the most stable and effective QTL and was centered on the *lfy1* interval. Furthermore, the additive effects showed that the leafy maize line Y915 increased LA at this locus (**Table 2**). These results prompt us to refer to *qLA3-4* as the *lfy1* gene. It is of interest that a QTL for LA was also detected at the *qLA3-4* region using a set of BC_2_S_3_ populations derived from normal maize line W22 and teosinte accession CIMMYT 8759 (Li D. et al., 2016). Further studies are needed to investigate whether the QTL identified in the maize-teosinte population is an allele of this gene or represents a linkage block containing the *lfy1* gene.

In addition to the major QTL *qLA3-4*, three LA QTL identified in the present study (*qLA 2-1*, *qLA 4-1*, and *qLA 8-1*) are also likely located in the same locations identified for LA in the maize-teosinte BC_2_S_3_ population (Li D. et al., 2016). However, these QTL were sensitive to the environment as they were detected in either only one or two environments in the present study. Furthermore, these QTL had relatively low LOD scores and explained a small proportion of the phenotypic variation in both studies. Therefore, these QTL can be regarded as minor QTL for LA.

The important QTL *qLA 7-1* detected in the F_2:3_ population, which was stable and explained a large proportion of LA phenotypic variation, was not detected in a maize-teosinte population (Li D. et al., 2016). Similarly, *qLA1-1*, an important QTL with a large effect on LA that was previously identified in the maize-teosinte population (Li D. et al., 2016), was not detected in the present study. Furthermore, the two major QTL together with the major QTL *qLA3-4* mentioned above were not detected in the GWAS. All of the QTL studies were based on natural genetic variation in the population under study. The inconsistency of these important QTL in the maize F_2:3_ population, maize-teosinte population and/or association population might be due to their different genetic backgrounds. These QTL might represent rare genes or alleles in the association mapping population. Alternatively, they could be confounded by the structure of the association population. GWAS has a poor strength to detect rare genes or genes that are confounded by a population structure (Yang et al., 2011). Other possible reasons might include environmental variation, experimental error, or statistical defects associated with gene mapping analysis (Zhao et al., 2011).

Significant correlations between LA and the days to tasseling and plant height were observed (**Figure 5**). Identification of QTL for several related traits allowed us to gain deeper insights into the relationships among the traits. The coincidence of QTL for two traits with allelic differences corresponding to the expected relationship between the traits is strong evidence that the two traits are causally related (Thumma and Naidu, 2001; Yin et al., 2010). Four LA QTL coincided with the QTL for the days to tasseling and/or plant height, and at each of the coincident QTL, the additive effect showed the same direction (**Figure 6**, **Table 2** and Supplementary Table S1). These results suggest a close relationship between LA and the days to tasseling and plant height. This conclusion is in contrast with a previous study showing that flowering time was not strongly related to LA (Li D. et al., 2016). LA had a higher correlation with the days to tasseling compared to plant height (**Figure 5**). This might be because the coincident QTL explained a higher phenotypic variation for the days to tasseling than for the plant height (**Figure 6**). Another possible reason is that there are additional “independent” plant height genes segregating the population of interest. These genes control the plant height but not LA. In agreement with the close relationship between LA and flowering time, at the major LA locus *qLA7-1* detected in our study, a QTL for flowering time was also revealed in a maize nested association mapping (NAM) population by Buckler et al. (2009).

Maize lines carrying the *lfy1* gene usually show late flowering times in addition to increased LA (Neuffer et al., 1997). Genetic evidence is lacking for the increased flowering time of the *lfy1* gene. In addition, little information on the effect of this gene on plant height is available. In the present study, the *lfy1* gene (*qLA3-4*) was found to have a significant effect on the days to tasseling and plant height (**Figures 7A–C**). It explained a phenotypic variation ranging from 27.1 to 29.9% for the days to tasseling and from 4.4 to 15.3% for the plant height in the F_2:3_ population (Supplementary Table S1). Furthermore, there was a significant difference in plant height and days to tasseling between the *lfy1* gene NILs, NIL^6L^ and NIL^9L^. These results suggest that the *lfy1* gene has a pleiotropic effect on LA, days to tasseling, and plant height. The co-association of a single gene with multiple traits that are phenotypically related has been reported in previous studies. For example, the vernalization genes *Vrn1*, *Rht-1* and *Rpd-1* in wheat have been shown to have multiple effects on agronomic traits (Yan et al., 2003; Eunjin et al., 2015).

A comprehensive analysis of the genomic region of *qLA3-4* and *qLA7-1* predicted two candidate genes, *GRMZM2G045275* and *GRMZM2G106903. GRMZM2G045275* encodes an early flowering protein in the *qLA3-4* region. The homolog of this candidate gene in rice is associated with the heading date, root development and kilo-grain weight (Fu et al., 2009). *GRMZM2G106903* encodes a flowering time control protein in the *qLA7-1* region. The homolog of this candidate gene in rice was involved in regulating plant height and flowering time (Chen et al., 2007). Because the above candidate genes have not been verified using transgenic methods and/or mutants, they may not be causal genes. As B73 is a normal line, the leafy trait gene might be missed in this line. Actually, the B73 reference genome may cover only ∼70% of the low-copy gene fraction of the maize inbred lines (Gore et al., 2009). Furthermore, non-coding sequences in the leafy maize may contribute to the leafy trait. Therefore, further map-based cloning, screening of the candidate regions from more BAC libraries, and verification of the causal genes for the major QTLs, *qLA3-4* and *qLA7-1*, are warranted.

The breeding of maize varieties with ideal architecture is an important tactic for the further improvement of grain production. QTL pyramiding is the process of combining several QTL from different loci for a specific trait to make superior genotypes (Xu, 1997). In the present study, QTL *qLA3-4* and *qLA7-1* were shown to have large effects on LA and were stably expressed across different environments (**Table 2**). These two QTL might be useful for the improvement of maize architecture. However, an increase of LA may increase water demand due to the increased leaf area (Lambert et al., 2014). High water demand is not a desirable trait in the drought stress condition. In arid and semi-arid areas, leafy maize may not grow well as normal maize. In contrast, in well-watered conditions the water demand is not a question for plant growth, and thus, the breeding of maize varieties with increased LA could provide a means for the development of ideal plant architecture and further improvement of grain yield. Considering that the *lfy1* gene increases not only LA but also plant height and flowering time, major genes for early maturity and/or reduced plant height should be employed in the development of leafy maize hybrids in areas where late-flowering time and tall plant height are unfavorable factors for maize production.

## Author Contributions

ZY, DD, and MC contributed to the study design; MC, BJ, HL, XK, YZ, RZ, ZL, and LY contributed to sample preparation, experimental execution and data analysis; BJ performed the bioinformatics analysis; and ZY and MC wrote the paper. All authors read and approved the final manuscript.

## Conflict of Interest Statement

The authors declare that the research was conducted in the absence of any commercial or financial relationships that could be construed as a potential conflict of interest.

## Supplementary Material

**FIGURE S1 |** Experimental flow chart for the construction of NILs. Y915 is a leafy inbred line which carries the *lfy1* gene. Y53 is a normal inbred line. After six generations of self-pollination of the F_1_ plants derived from Y915 and Y53, two F_8_ homozygous inbred lines were developed from two F_7_ individuals of a single F_6_ plant. The two homozygous inbred lines, designated NIL^6L^ and NIL^9L^, stably express 6 and 9 LA, respectively. The gray and white colors represent individuals that are segregating and non-segregating for LA in their progenies, respectively. The circles and triangles represent leafy and normal materials, respectively.

**FIGURE S2 |** Linkage maps based on genotyping of F_2_ population. Numbers to the left indicate linkage distance; marker names are given to the right of each chromosome.

**FIGURE S3 |** Linkage disequilibrium (LD) analysis using the *R*^2^ correlation between each marker within the association hot spots. Solid black lines represent the LD blocks.

**FIGURE S4 |** Genomic background of the two NILs. The 10 maize chromosomes are indicated by black bars. The red bar represents the *qLA3-4* region. The red lines represent polymorphic SSR markers between the two NILs.
