# Supplementary figures and images for: Genetic Mapping of the Leaf Number above the Primary Ear and Its Relationship with Plant Height and Flowering Time in Maize
Source: Front Plant Sci. 2017 Aug 18;8:1437. doi: 10.3389/fpls.2017.01437 (PMC5563357; doi:10.3389/fpls.2017.01437)

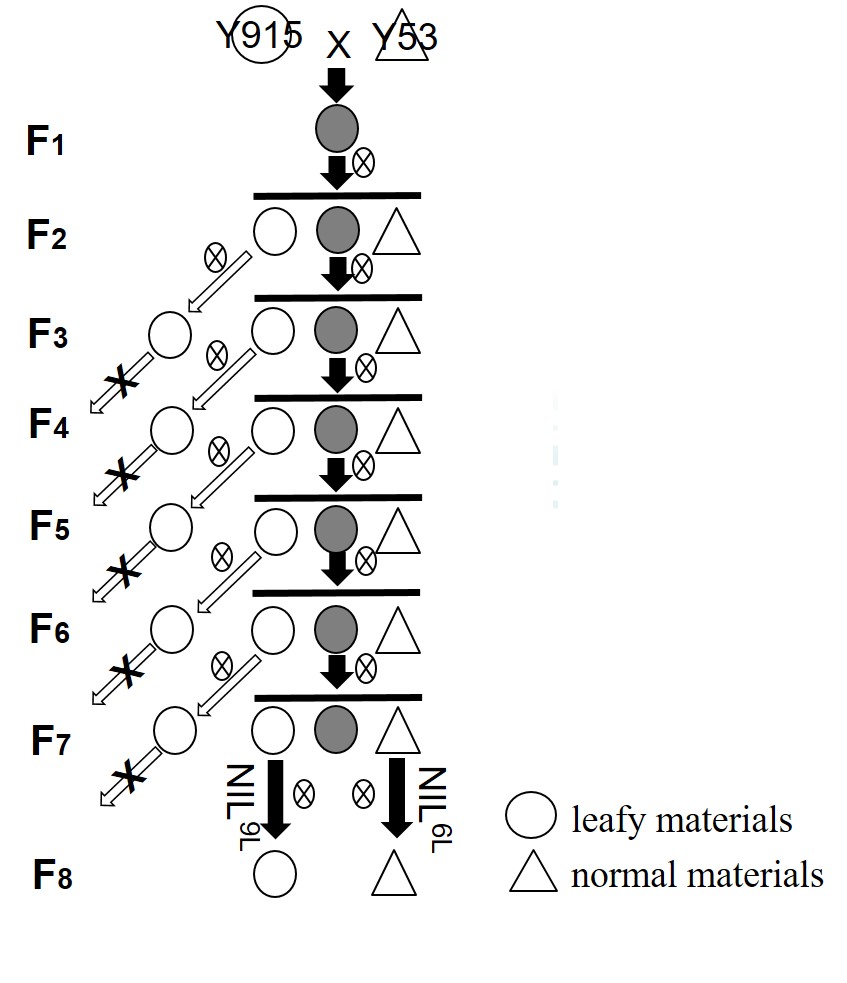

Supplement: Supplementary file 1 [file Image_1.JPEG]

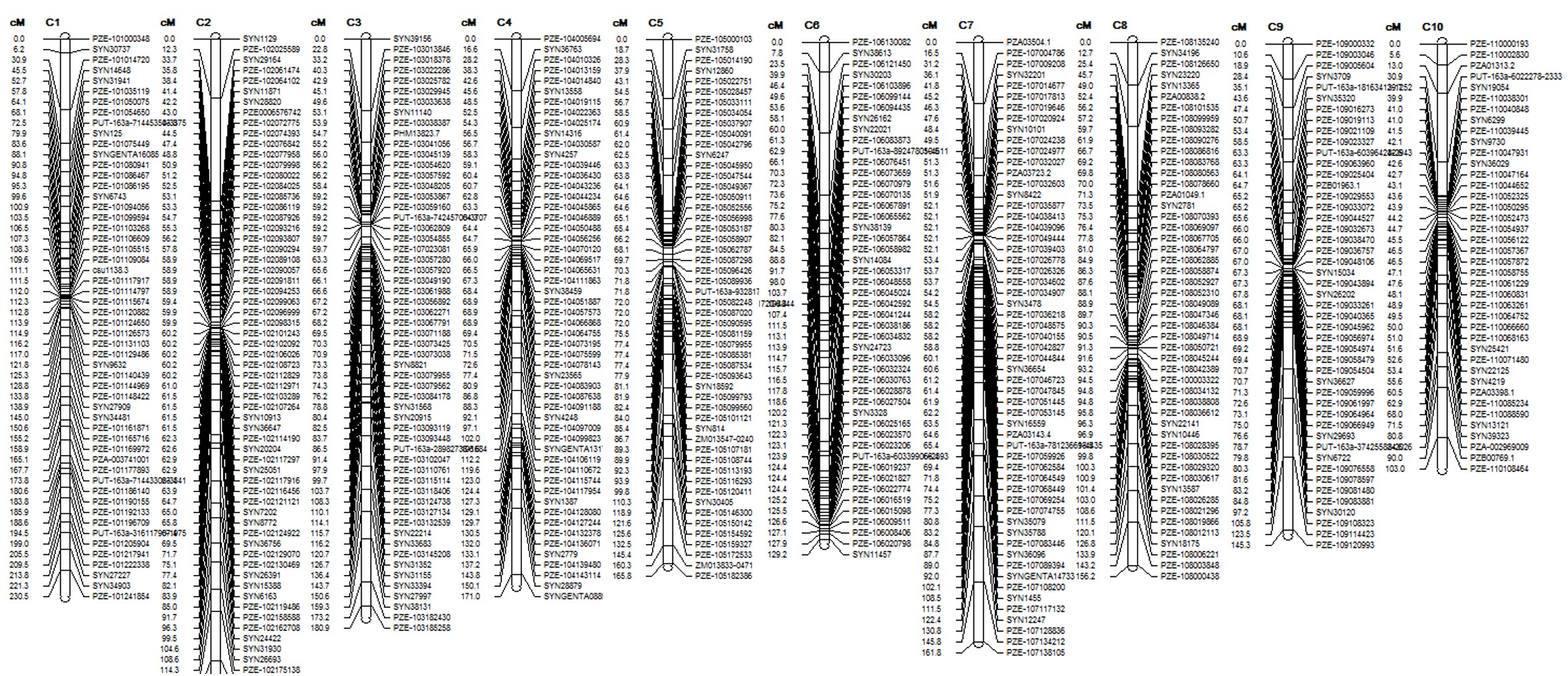

Supplement: Supplementary file 2 [file Image_2.JPEG]

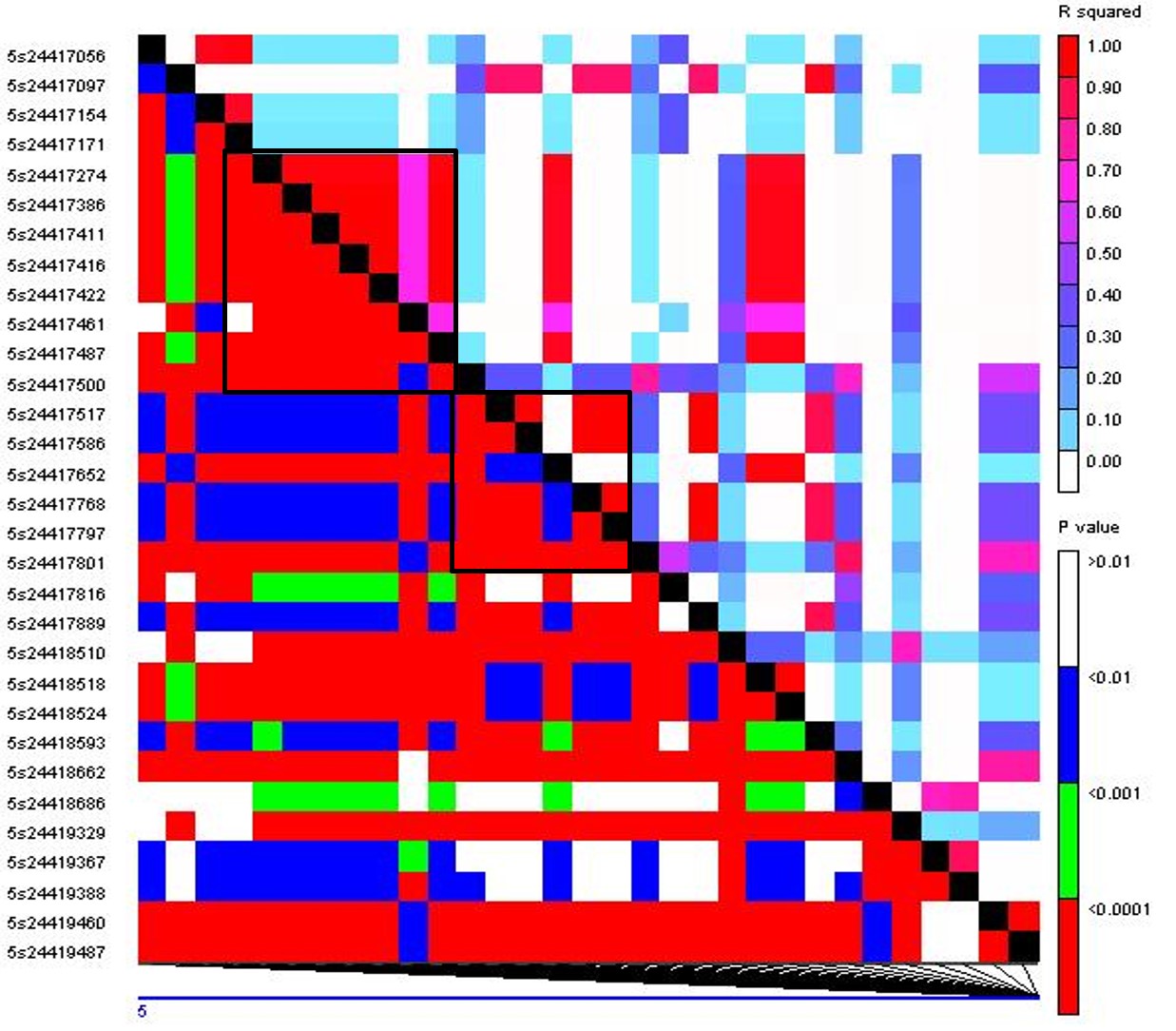

Supplement: Supplementary file 3 [file Image_3.JPEG]

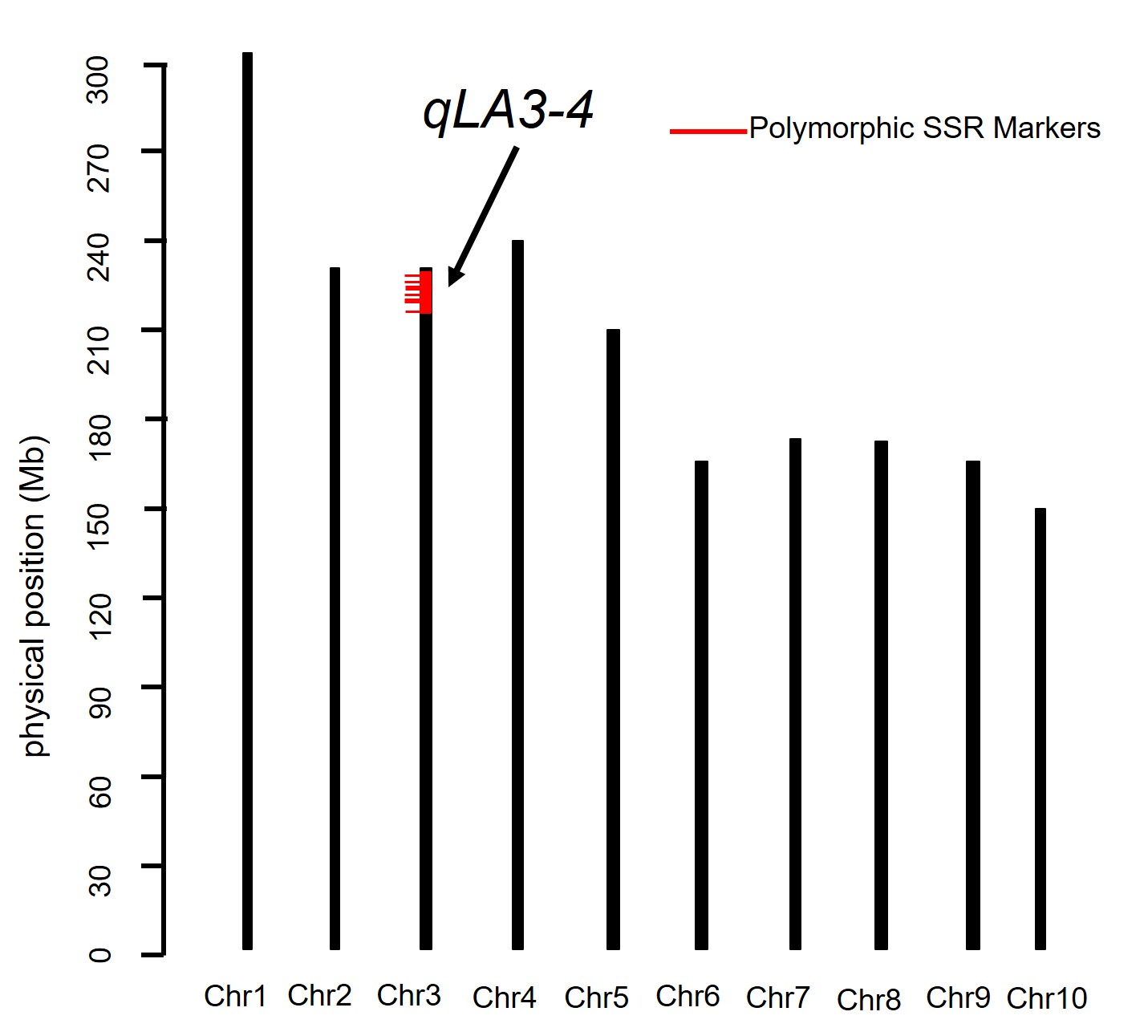

Supplement: Supplementary file 4 [file Image_4.JPEG]
