# Supplementary material for: Genetic Mapping of the Leaf Number above the Primary Ear and Its Relationship with Plant Height and Flowering Time in Maize
Source: Front Plant Sci. 2017 Aug 18;8:1437. doi: 10.3389/fpls.2017.01437 (PMC5563357; doi:10.3389/fpls.2017.01437)
Supplement: Supplementary file 5 [file Table_1.DOCX]

| **Table S1 \| Quantitative trait loci for plant height (PH) and days to tasseling (DTT) detected in different environments** | | | | | | | | | | | | | | | | |
| --- | --- | --- | --- | --- | --- | --- | --- | --- | --- | --- | --- | --- | --- | --- | --- | --- |
| **Traits** | **QTL** | **Chr** | **Marker interval** | **Position(cM)** | **Yangzhou** | | | | **Huai’an** | | | | **Hainan** | | | |
|  |  |  |  |  | **LOD** | **Add^a^** | **Dom^b^** | ***R^2c^* (%)** | **LOD** | **Add** | **Dom** | ***R^2^* (%)** | **LOD** | **Add** | **Dom** | ***R^2^* (%)** |
| PH | *qPH 1-1* | 1 | PZA00393.1 - ZM012984-0229 | 12.8-24.9 |  |  |  |  |  |  |  |  | 3.60 | 4.75 | -1.06 | 0.9 |
|  | *qPH 1-2* | 1 | PZE-101192454- PUT-163a-71445350-3375 | 71.2-72.5 | 2.73 | -0.25 | 10.37 | 3.1 |  |  |  |  |  |  |  |  |
|  | *qPH 3-1* | 3 | SYN33394- PZE-103166902 | 143.7-144.9 | 2.93 | 12.61 | 0.18 | 5.3 |  |  |  |  |  |  |  |  |
|  | *qPH 3-2* | 3 | PZE-1031727322-PZE-103179063 | 153.4-164.4 | 6.63 | -20.41 | -1.18 | 10.9 | 8.15 | -13.45 | 0.55 | 15.3 | 2.50 | -1.18 | -5.09 | 4.4 |
|  | *qPH 5-1* | 5 | PZE-105094820 -PZE-105105785 | 77.9-88.3 |  |  |  |  |  |  |  |  | 3.19 | -4.91 | 2.45 | 11.1 |
|  | *qPH 5-2* | 5 | PZE-105105785 – PZE-105120411 | 89.3-99.7 |  |  |  |  | 4.53 | -9.08 | 3.48 | 9.9 |  |  |  |  |
|  | *qPH 5-3* | 5 | PZE-105150142- PZE-105157516 | 121.6-130.6 | 6.19 | -11.67 | 6.57 | 18.4 |  |  |  |  |  |  |  |  |
|  | *qPH 6-1* | 6 | SYN11200 - PUT-163a-78119421-4381 | 13.9-20.5 |  |  |  |  | 4.08 | 9.02 | -0.60 | 7.4 | 5.95 | 6.62 | -2.05 | 19.9 |
|  | *qPH 6-2* | 6 | PZE-106054189- PZE-106041977 | 90.7-107.1 |  |  |  |  |  |  |  |  | 2.70 | -3.08 | 3.62 | 8.5 |
|  | *qPH 7-1* | 7 | SYN13918 - PZE-107068449 | 54.2-74.4 | 2.92 | 7.72 | 1.24 | 4.6 | 7.48 | 11.98 | -5.48 | 17.8 |  |  |  |  |
|  | *qPH 9-1* | 9 | PZE-109085150- SYN30120 | 86.4-97.1 | 2.79 | -4.15 | 9.62 | 6.5 |  |  |  |  |  |  |  |  |
| DTT | *qDTT 1-1* | 1 | SYN29751-PZE-101163212 | 130.3-151.7 | 3.08 | -1.29 | -0.59 | 2.2 |  |  |  |  |  |  |  |  |
|  | *qDTT 1-2* | 1 | ZM013506-0433-PZE-101205159 | 195.0-196.9 |  |  |  |  | 2.57 | 0.62 | 1.22 | 2.9 |  |  |  |  |
|  | *qDTT 3-1* | 3 | SYN39156-SYN170 | 0-13.2 |  |  |  |  |  |  |  |  | 3.15 | 0.33 | 1.46 | 5.2 |
|  | *qDTT 3-2* | 3 | PZE-103033638 - PZE-103072066 | 49.6-71.7 |  |  |  |  | 3.88 | 1.33 | -0.25 | 5.5 |  |  |  |  |
|  | *qDTT 3-3* | 3 | PZE-103132539- PZE-103142654 | 118.2-122.8 |  |  |  |  |  |  |  |  | 2.93 | 0.42 | -1.31 | 5.4 |
|  | *qDTT 3-4* | 3 | SYN32259- PZE-103182430 | 148.9-173.2 | 23.79 | -4.29 | -4.29 | 28.6 | 25.57 | -4.01 | -1.21 | 27.1 | 20.61 | -3.42 | -1.09 | 29.9 |
|  | *qDTT 5-1* | 5 | PZE-105108744 - PZE-105116293 | 89.9-93.9 |  |  |  |  | 3.12 | -0.69 | -1.18 | 3.6 |  |  |  |  |
|  | *qDTT 6-1* | 6 | PZE-106048558- PZE-106045024 | 97.9-103.7 |  |  |  |  | 3.06 | 0.90 | 0.90 | 1.6 | 3.75 | 0.73 | 1.30 | 5.9 |
|  | *qDTT 7-1* | 7 | PZE-107020924-SYN21763 | 47.6-71.2 | 4.80 | 0.73 | -1.99 | 7.6 | 5.37 | 1.69 | -0.83 | 11.1 | 4.70 | 1.33 | -0.89 | 10.8 |
|  | *qDTT 7-2* | 7 | PZE-107133122-PZE-107134212 | 144.5-145.8 | 2.59 | -1.17 | 0.33 | 4.6 |  |  |  |  |  |  |  |  |
| ^a^ Positive value indicates the increasing effects contributed by Z58, and negative value indicates the increasing effect contributed by Y915 in the F_2:3_ population.  ^b^ Positive values of the dominance effect indicate that the heterozygotes have higher phenotypic values than the respective means of two homozygotes. Negative values indicate that heterozygotes have lower phenotypic values than the respective means of two homozygotes.  ^c^ Percentage phenotypic variation explained by QTL. | | | | | | | | | | | | | | | | |
