# Supplementary material for: Genetic Mapping of the Leaf Number above the Primary Ear and Its Relationship with Plant Height and Flowering Time in Maize
Source: Front Plant Sci. 2017 Aug 18;8:1437. doi: 10.3389/fpls.2017.01437 (PMC5563357; doi:10.3389/fpls.2017.01437)
Supplement: Supplementary file 6 [file Table_2.DOCX]

| **Table S2 \| Quantitative trait locus correspondence possibility expected by chance, ** Significant at *p < 0.01*; * Significant at *p < 0.05.*** | | |
| --- | --- | --- |
|  | LA | PH |
| PH | 0.0470* |  |
| DTT | 0.0366* | 0.0067** |
| LA, leaf number above the primary ear; PH, plant height; DTT, days to tasseling. | | |
